# Haemangioblastomatosis

**DOI:** 10.5334/jbr-btr.839

**Published:** 2015-09-15

**Authors:** P. Gillardin, M. Lemmerling, F. Bouttens, H. Colle, R. De Potter, A. I. De Backer

**Affiliations:** 1 Department of Radiology, AZ Sint-Lucas, Ghent, Belgium; 2 Department of Radiology, UZ Gasthuisberg, Leuven, Belgium; 3 Department of Oncology, AZ Sint-Lucas, Ghent, Belgium; 4 Department of Neurosurgery, AZ Sint-Lucas, Ghent, Belgium; 5 Department of Neurology, AZ Sint-Lucas, Ghent, Belgium

A 56-year-old female, known with a meningioma and history of histologically confirmed cerebellar hemangioblastoma, was referred to our department with complaints of equilibrium disturbances, stomach aches, nausea and vomiting. Brain MRI evaluation was performed and showed a status after surgical excision of the hemangioblastoma in addition to a known meningioma. Besides these expected findings, multiple foci of contrast-enhancing, nodular leptomeningeal thickening were present, dispersed through the posterior fossa and around the brain stem. Meningeal thickening was also prominently present in the internal auditory canal on the left. Axial postgadolinium T1-weighted image shows pathologic enhancement in the posterior fossa at the level of the pontocerebellar cistern and internal auditory canal on the left, expanding in the prepontine cistern and the internal auditory meatus (Fig. A, arrows) and in the paradural region bilaterally. Furthermore, disseminated perimedullary involvement of the cervical spine was observed (Fig. B). Sagittal postgadolinium T1-weighted images shows nodular enhancing lesions along the leptomeninges of the cervical, dorsal and lumbar spine (arrows). Full spine MRI, revealed additional lesions in the thoracic and lumbar spine, suggestive of drop lesions (Fig. C).

**Figures A–C:**
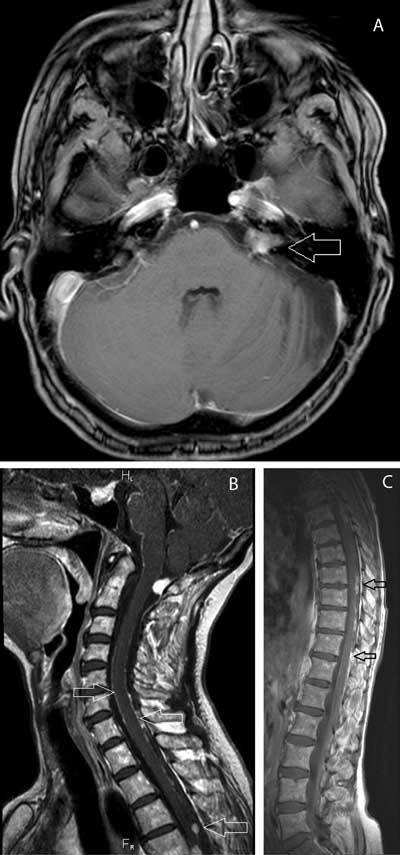


The lesion in the left pontocerebellar region was resected and pathologic report confirmed the diagnosis of hemangioblastomatosis. The patient was discharged with persistent left hearing impairment. Genetic testing for Von Hippel Lindau was requested, as no other associated lesions were clinically present.

## Comment

Haemangioblastomatosis is an exceptional, hypervascular, meningeal neoplasm of the central nervous system. This benign tumoral entity, which consists of multifocal, recurrent haemangioblastomas, usually involves the brain and spinal cord. The disease can be associated with Von Hippel-Lindau (VHL) syndrome (38%) but more often, it occurs sporadically (62%) [1]. Up to 95 % of the disseminated encephalic haemangioblastomas arise in the posterior fossa, with the cerebellar hemispheres as predisposing site. This infratentorial dominance of the lesion localization is believed to be the result of gravity and physiologic CSF flow. No case of haemangioblastomatosis has been reported without prior surgical intervention, supporting the hypothesis that CSF spillage could be etiologic to disseminated hemangioblastomas.

Other known localizations of haemangioblastomas are the retina, brain stem and spinal cord. Supratentorial localisation is more common in VHL setting, besides the classically associated pheochromocytoma, renal cell carcinoma or cystic lesions of the pancreas or kidney. Spinal hemangioblastomas are mainly found posterior to the cord. They appear as well defined, contrast enhancing lesions, layering the leptomeninges.

Haemangioblastomatosis, or scattered haemangioblastomas, is very rare. Presence of VHL-like pathology, and additional genetic and histopathologic confirmation, can facilitate the differential diagnosis with metastasis, pilocytic astrocytoma or schwannomas. Adequate measures should be considered during interventions to minimalize CSF spillage, as outcome after recurrence is usually poor.

## Competing Interests

The authors declare that they have no competing interests.
